# Contrasting the impact and cost-effectiveness of successive intervention strategies in response to Ebola in the Democratic Republic of the Congo, 2018–2020

**DOI:** 10.1136/bmjgh-2024-015822

**Published:** 2025-04-15

**Authors:** Thibaut Jombart, Wu Zeng, Michel Yao, Anne Cori, Steve Ahuka-Mundeke, Hadia Samaha, Thomas Wilkinson, Mathias Mossoko, Jean-Pierre Lokonga, Dominique Baabo, Fatima El Kadiri El Yamini, Patrick Hoang-Vu Eozenou, Sylvain Yuma, Linda Mobula

**Affiliations:** 1 MRC Centre for Global Infectious Disease Analysis, Department of Infectious Disease Epidemiology, School of Public Health, Imperial College London, London, UK; 2 Department of Global Health, Georgetown University, Georgetown University, Washington, District of Columbia, USA; 3 World Health Organization, Geneva, Switzerland; 4 MRC Centre for Global Infectious Disease Analysis, Jameel Institute, School of Public Health, Imperial College London, London, UK; 5 Institut National de Recherche Biomédicale, Kinshasa, Democratic Republic of Congo; 6 Health, Nutrition and Population, World Bank Group, Washington, District of Columbia, USA; 7 Ministry of Health, Kinshasa, Democratic Republic of Congo; 8 Health System Development Project, Kinshasa, Democratic Republic of Congo; 9 UNICEF, Kinshasa, Democratic Republic of Congo

**Keywords:** Mathematical modelling, Viral haemorrhagic fevers, Health systems evaluation

## Abstract

**Introduction:**

The 10th outbreak of Ebola Virus Disease (EVD) in the Democratic Republic of the Congo (DRC) in 2018–2020 was the largest in DRC’s history and the second largest worldwide. Different strategic response plans (SRPs) were implemented, and the outbreak was eventually stopped after a large scale-up of operations with the SRP 4, which benefited from all public health measures deployed during SRPs 1-3, upon which it developed a more holistic approach including community engagement, logistics and security.

**Methods:**

We used modelling to characterise EVD transmission and assess the epidemiological impact of the two main response strategies (SRPs 1–3 vs SRP 4). We simulated potential future epidemics with different intervention scenarios, combined with a costing model to evaluate the incremental cost-effectiveness of different strategies.

**Results:**

We estimated a mean effective reproduction number *R* of 1.19 (credible interval (95% CrI) = (1.13 ; 1.25)). The spatial spread was moderate with an average 4.4% (95% CrI = (3.5%; 5.4%)) of transmissions moving to different health zones. The scale-up of operations in SRP 4 coincided with a threefold reduction in transmission, and 30% faster control of EVD waves. In simulations, SRP 4 appears cost-saving, although most simulated outbreaks remain small even with SRPs 1–3.

**Conclusion:**

Most EVD outbreaks are expected to be small and can be contained with SRPs 1–3. In outbreaks with increased transmissibility or in the presence of insecurity, rapid scale-up to SRP 4 is likely to save lives and be cost-effective.

**WHAT IS ALREADY KNOWN ON THIS TOPIC**

- The 10th outbreak of Ebola Virus Disease (EVD) in the Democratic Republic of the Congo (DRC) in 2018–2020 was the largest in DRC’s history.
- The overall impact and cost-effectiveness of the response to this epidemic remain unclear.

**WHAT THIS STUDY ADDS**

- We estimate the transmissibility, dispersal intensity and response efficacy for this outbreak.
- A holistic response strategy which included not only public health counter-measures but also community engagement, logistics and security was accompanied by a threefold reduction in EVD transmission.
- We provide a costing model for response activities around EVD cases, and estimates of the cost-effectiveness of the different response strategies.

**HOW THIS STUDY MIGHT AFFECT RESEARCH, PRACTICE OR POLICY**

- Our results suggest that large-scale, holistic approaches to EVD response, while requiring initially higher investments, not only save lives but are also likely cost-effective in the long run.

## Introduction

 The 10th outbreak of Ebola Virus Disease (EVD) in the Democratic Republic of the Congo (DRC) in 2018–2020 was the largest in DRC’s history, and the second largest EVD epidemic globally.^1^ With a total of 3481 reported cases and 2299 deaths,^2^ this epidemic was located in the North Kivu and Ituri provinces, a fragile environment in which recurrent insecurity events had a direct impact on transmission patterns and on response activities.^3^,^7^ As a consequence, response strategies evolved in the course of the outbreak to adapt to the complexities of deploying interventions amidst the ever-changing challenges of an armed conflict area.

The international response led by DRC’s Ministry of Health involved a wide range of partners including the United Nations agencies and international non-governmental organisations (NGOs). This response was planned and coordinated through strategic response plans (SRPs), which laid out plans for response activities and implementation by different partners and were used to calculate the budget of the response. Interventions to stop EVD outbreaks typically involve a range of activities including active case detection and testing, patient isolation and treatment, contact tracing, ring vaccination, safe and dignified burials to avoid funeral exposures, and prevention.^1 8 9^ SRPs had four consecutive iterations (SRP 1–SRP 4), with the first three (SRPs 1–3) focusing purely on public health responses (detailed below), after which strategies evolved to a multisectoral/humanitarian response under SRP 4^10^ (see also online supplemental tables S1 and S2).

SRPs 1–3 were developed in a context where the provinces of North Kivu and Ituri were experiencing their first Ebola outbreak. It was therefore important to focus efforts on building local capacity of national staff and NGO partners, to take into account innovations from previous outbreaks in case management, infection prevention and control, ring vaccination, and community engagement (dialogue, community leaders rapid intervention group) for community-based interventions in the Ebola response. These response plans also helped in implementing measures to address challenges related to the context, including insecurity, community reluctance, and humanitarian needs.

Distrust in communities, population displacement and worsening insecurity affected negatively the response,^3^,^511^ culminating in the destruction of the Ebola treatment centres (ETCs) of Katwa and Butembo in late February 2019, which led to an increase in transmission and flare-ups of cases from March to May 2019.^5^ These persistent difficulties in controlling the outbreak resulted in a step-change with the SRP 4, which was implemented on the 15 July 2019 (438 days into the outbreak). Building on structures set by and lessons learnt from SRPs 1–3, SRP 4 was designed to address existing gaps, and build on previous successes with a more experienced team for final containment. This approach hypothesised that delivering integrated interventions, tailored to the context—especially considering humanitarian challenges and insecurity—while scaling up all EVD-related actions in close collaboration with communities, would achieve greater impact. This new strategy involved a significant scale-up of operations with a 10-fold increase of budget, in which the multisectoral/humanitarian response included not only activities pertaining to the public health response but also other key activities to address challenges posed by insecurity and community reticence, which can be broken down into four pillars of intervention.

Pillar one was focused on the public health response itself, including improved community-based detection of cases, and deployment of rapid response teams (benefitting from local staff trained during SRPs 1–3) in areas with no Ebola case reported to facilitate immediate responses in the event of new flare-ups of cases. This approach permitted the deployment of multisectoral interventions within 24–48 hours of case detection, where reinforced contact identification and follow-up, in-depth investigations of reported contacts, and setting up community networks around cases facilitated the rapid detection of secondary cases.

The second pillar pertained to security and logistics for the overall response, which represented key challenges in the context of mounting insecurity. Pillar 3 aimed at strengthening risk communication and community engagement, by fostering a dialogue with affected communities, and considering their needs beyond Ebola, which was not usually perceived as a priority. This included activities such as water, sanitation and hygiene (WASH) interventions and cash for work interventions that included risk communication messages on Ebola and other diseases. Finally, pillar 4 was dedicated to regional preparedness for future outbreaks and was not directly part of the EVD response in DRC.

While the epidemic was eventually contained under the SRP 4, no evaluation of the impact and cost-effectiveness of the different response strategies has been made to date. To fill this gap, we evaluated the impact of interventions, by comparing EVD transmission as described by a Bayesian meta-population branching process model, during the SRPs 1–3 and SPR 4 strategies. Using simulations, we evaluate the potential impact of the different public health strategies that could be used in future Ebola responses, contrasting SRPs 1–3 to the strategy used under SRP-4. Building upon previously collected cost information on various EVD control and management interventions for this epidemic, we assess the cost-effectiveness of the different response strategies.

## Methods

### Data

We used a linelist describing key information for a total of 3470 reported cases for which data were available, extracting the dates of onset, disease outcome and health zone in which the cases were reported (level 1 administrative location) for every case. 37 cases for which dates of onset or health zones were missing were removed. The resulting dataset comprised 3433 cases, including 2262 deaths (1152 survivors, 19 unknown outcomes), distributed over 29 affected health zones, and spanning 719 days from the first (3 May 2018) to the last reported case (21 April 2020). In the absence of sufficient data to estimate under-reporting,^12^ we assumed constant reporting throughout the epidemic. Distinct epidemic waves were identified for each health zone using temporal clustering,^13^ considering two successive cases as belonging to different waves if their dates of symptom onset were more than a serial interval apart (online supplemental text S1). The resulting clusters were manually refined to distinguish successive waves separated by a dip in incidence. Each data point was then classified into three categories: ‘growth’ phase (before the peak of the wave), and ‘control’ phase (*ie*, after the peak) during SRPs 1–3 or SRP 4.

In addition, the ‘delay to control’, defined as the number of days between the first reported case and the peak of a wave, was also calculated for every wave in every health zone, and used to fit separate discretised gamma distributions for SRPs 1–3 and SRP 4.^14^ Waves overlapping both periods were assigned to the period in which they had most days.

### Estimation of epidemiological parameters

We used a Bayesian meta-population, branching process model to describe the transmission of EVD in different health zones (online supplemental text S2). Each health zone was treated as a separate population, with a homogeneous diffusion of transmission among all 29 populations. The model likelihood was defined as the probability of observing daily incidence in the different health zones given past case incidence, as a function of four different parameters: the effective reproduction number *R* during growth phases, defined as the average number of secondary cases per infected patient, the dispersal *ρ* representing the proportion of the force of infection directed towards other populations (health zones), and strategy efficacies describing the relative reduction of transmission during control phases during SRPs 1–3 (*ε*_SRP1-3_) or SRP 4 (*ε*_SRP4_). Note that here, *R* is not a basic reproduction number, as it already includes the impact of interventions before the point of control. The impact of the depletion of susceptible individuals, deemed negligible here due to a very small final attack rate (<0.0005), was not included in the model.

*R*, *ρ*, *ε*_SRP1-3_ and *ε*_SRP4_ were all estimated from the data. Because of the rather exceptional setting of the outbreak in North Kivu/Ituri, where insecurity had a key impact on EVD transmission,^13^,^5^ we used flat, ‘uninformative’ priors for all parameters, so that the posterior distribution was directly proportional to the likelihood. Markov chain Monte Carlo (MCMCs) were used to explore the parameter space and obtain posterior samples for each parameter (online supplemental text S3).

### Forward simulations

To assess the potential impact of different intervention strategies, we used our calibrated model to simulate potential outbreaks under different settings. All simulations were run for 2 years with 50 patches, started with a single case, and reported new daily cases in every patch. For each simulation, we calculated monthly numbers of cases, deaths (using the mean case fatality ratio estimated from the data) and patches affected. We further classified patches according to the following categories: *inactive* (has not seen cases yet), *active* (with at least one case in the last 2 months), in ‘*watching mode*’ (no longer active, but with at least one case in the last 3 months) and *deactivated* (no longer ‘active’ or ‘watching’), to acknowledge the different costs associated with different levels of alert. Monthly patch counts were further broken down into these four categories. We also reported the total numbers of cases and patches affected (*ie*, *active* at least once) for each outbreak.

Two different types of scenarios were simulated. In the first, we contrasted responses where only SRPs 1–3 or SRP 4 strategy was used, using values of *R*, *ρ* and the strategy efficacies (*ε*_SRP1-3_ and *ε*_SRP4_), as well as delays to control estimated from the data. 10 000 independent replicates were generated for each strategy, using parameters drawn randomly with replacement from the joint posterior samples of our MCMCs, resulting in 20 000 simulated outbreaks.

In a second set of simulations, we evaluated the impact of implementing the SRP 4 strategy at various delays after initiation of the SRPs 1–3. In this scenario, SRPs 1–3 were implemented 4 weeks after the first case, and the SRP 4 strategy was then deployed 12, 24, or 48 weeks after the SRPs 1–3. 10 000 independent replicates were generated for each delay, resulting in 30 000 simulations.

### Cost-effectiveness estimation

Cost-effectiveness analyses were conducted for each simulation. Unit costs of key interventions are listed in table 1. Cost estimates for a given simulation were obtained by multiplying relevant unit costs by the numbers of cases, deaths and active patches and the duration of the outbreak. Disability-adjusted life years (DALYs) were also calculated for each scenario (online supplemental text S4).

**Table 1:** Unit cost of key interventions for Ebola Virus Disease control

Intervention	Unit cost (US$)
Rapid response team (per team per month)	66 182.00
Contact tracing and surveillance (per identified case)	4435.00
Laboratory test (per identified Ebola case)	4166.00
Management of confirmed cases in Ebola treatment centre (per case)	1464.00
Management of suspected cases in Ebola treatment centre (per case)	358.38
Management of confirmed cases in Ebola transit centre (per case)	219.28
Management of suspected cases in Ebola transit centre (per case)	220.01
Vaccination (per vaccinated individual)	120.70
Psychosocial support (per beneficiary)	175.00
Prevention, coordination, community engagement, operation, and monitoring and evaluation (per capita per month)	2.71
Security+regional preparedness (per person per month) SRP 4 only	4.38

Source: Zeng *et al.*^10^

We also conducted a sensitivity study on unit costs per EVD case, disability weights and duration of illness. When a SD could not be estimated from the data, we assume it to be 20% of its corresponding mean.^15^ Gamma distributions were used for the parameters for costs, ratios and duration of illness, while beta distributions were used for parameters on disability weights. These distributions were used to generate 10 000 independent sets of parameters, each providing a different estimate of incremental costs and incremental effectiveness (DALYs averted). We calculated the mean of incremental costs and incremental effectiveness and their 95% quantile intervals.

## Results

### Characterising EVD transmission

**Figure 1:**
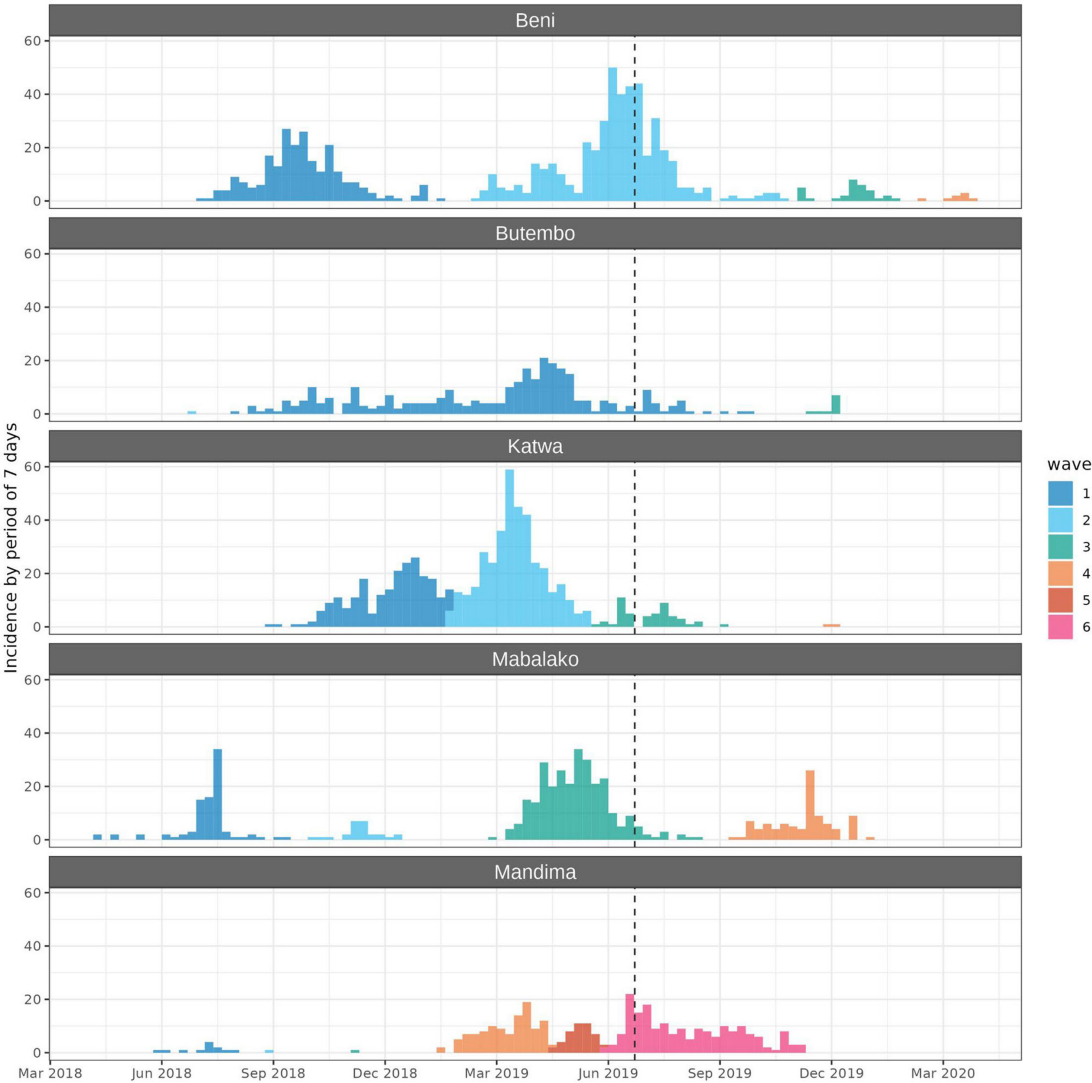
Epidemic curves of the five most affected health zones. This graph shows the weekly incidence by date of symptom onset of the five health zones with most reported cases. Colours indicate the different waves in each health zone. As waves were identified from daily incidence, some weeks may include overlapping waves. The vertical dashed line marks the change of strategy from SRPs 1–3 to SRP 4, on 15 July 2019.

Health zones with most EVD cases were characterised by successions of up to 6 waves of infection, including several waves after the SRP 4 was implemented (figure 1). The case fatality ratio was 66.2% (95% CI = (64.6%; 67.8%)), in line with previous outbreaks.^8 9^ Delays to control of the different waves were substantially longer in SRPs 1–3 (mean: 24.0 days) than in SRP 4 (mean: 17.5 days), although both distributions showed large variability (sd_SRP1-3_: 34.4 days; sd_SRP4_: 24.3 days) and were highly skewed (online supplemental figure S1, online supplemental table S4).

**Figure 2:**
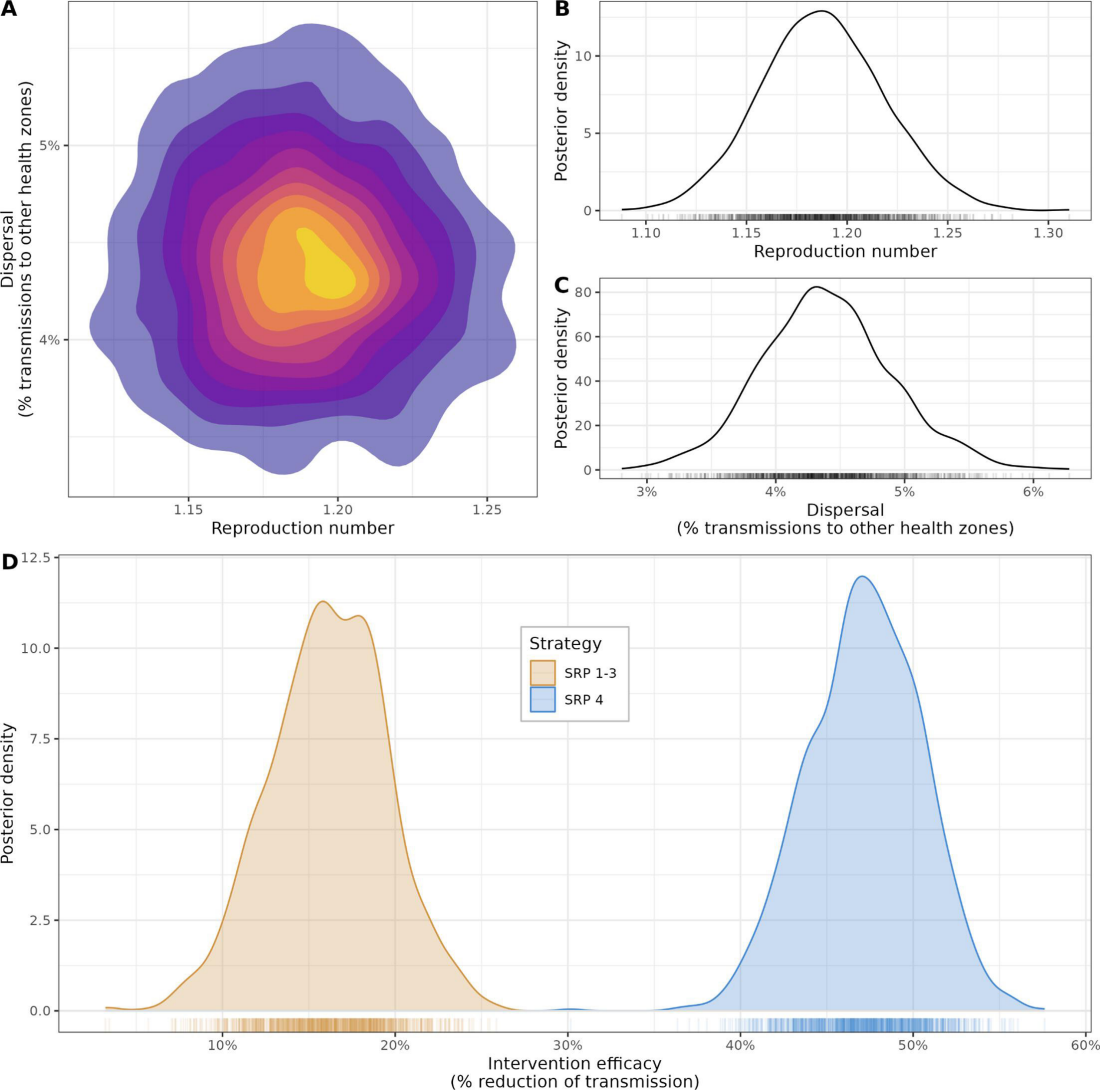
Estimation of transmission parameters of Ebola Virus Disease in North Kivu/Ituri. This figure summarises the estimation of the model parameters using MCMCs, using 1960 posterior samples after removing burn-in. (**A**) Posterior density of the joint distribution of the reproduction number (*R*) during *growth* phases and the dispersal parameter (*ρ*) expressed as the percentage of infections moving to different health zones; warmer colours indicate higher density. (**B**) Marginal posterior distribution of the reproduction number *R*. (**C**) Marginal posterior distribution of the dispersal parameter *ρ*. (**D**) Marginal distributions of the strategy efficacies for SRPs 1–3 and SRP 4, expressed as per cent reduction of *R* during the *control* phases.

MCMC chains showed rapid mixing after the first 100 iterations, discarded as burn-in (online supplemental figure S2) resulting in a posterior sample size of 1960. The clearly non-uniform posterior density surface suggested the data were highly informative about plausible values of *R* and *ρ*, with no evidence of correlation between transmission and dispersal (figure 2A). The average estimate of *R* during growth phases was 1.19 (figure 2B), with relatively little uncertainty (median: 1.18; 95% credibility interval (95% CrI) = (1.13; 1.25)). Dispersal across health zones (*ρ*) was limited (figure 2C), with an average 4.4% of transmission directed towards other health zones (median: 4.4%; 95% CrI = (3.5%; 5.4%)). Strategy efficacies clearly differed between strategies (figure 2D), with a relative reduction of transmission averaging 16.0% during SRPs 1–3 (median: 16.1%; 95% CrI = (9.3; 22.7)), compared with 46.8% during SRP 4 (median: 46.7%; 95% CrI = (40.7%; 53.2%)). This translated into effective *R* during decline phases averaging 1.00 during SRPs 1–3 (95% CrI = (0.94; 1.05)), compared with an average of 0.63 (95% CrI = (0.56; 0.69)) for SRP 4. Simply put, the reduction in transmission during SRP 4 was about three times higher than that during SRPs 1–3 (mean: 3.0; 95% CrI = (2.1; 4.8)).

### Simulating the impact of future interventions

**Figure 3:**
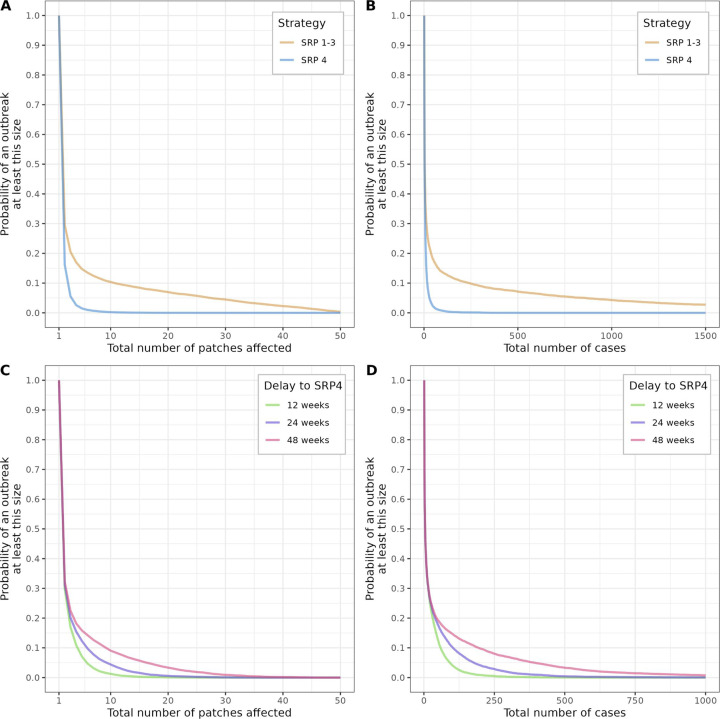
Results of simulated Ebola Virus Disease (EVD) outbreaks. Summary of simulated EVD outbreaks under two scenarios, first contrasting strategic response plan (SRP) 1–3 and SRP 4 strategy (**A, B**), then studying the impact of different delays to implementing SRP 4 after SRPs 1–3 (**C, D**). Results are reported as probabilities of simulated outbreaks being at least certain sizes, expressed as total number of patches affected out of a maximum of 50 (**A, C**), and as total number of cases (**B, D**). Each curve was derived from 10 000 simulations.

Simulated epidemics in which either SRPs 1–3 or SRP 4 would be employed showed similar patterns of heterogeneity in outbreak sizes, where a large number of small outbreaks were observed, alongside a few, much larger outbreaks (figure 3A,B). For instance, the proportions of outbreaks with at least 10 cases for SRPs 1–3 and SRP 4 were 33.3% and 19.0%, respectively. Clear differences between the two strategies were observed for larger epidemics, which were much more frequent under SRPs 1–3 than under SRP 4, both in terms of geographic spread (figure 3A) and total number of cases (figure 3B). For instance, outbreaks of at least 100 cases were 20 times more likely to occur under SRPs 1–3 than under SRP 4; this ratio increased to more than 700 when considering outbreaks of at least 500 cases (figure 3B).

We also evaluated the potential impact of implementing SRP four at various delays after SRPs 1–3. A large heterogeneity in outbreak size distribution was, again, observed across the board, with most outbreaks remaining limited, and a few, much larger outbreaks (figure 3C,D). Clear differences appeared across the different delays to SRP 4 implementation, with larger outbreaks, both in terms of geographic spread (figure 3C) and total numbers of cases (figure 3D) being observed when increasing the delay to SRP 4. For instance, the probability of observing an outbreak of at least 100 cases, smallest with a delay of 12 weeks (4.2% of simulations), increased by a factor of 2.5 for a delay of 24 weeks, and by a factor of 3.5 for 48 weeks. As expected, due to the exponential nature of epidemic growth, this effect was also non-linear: the probability of observing an outbreak of at least 500 cases, lowest for a delay of 12 weeks (0.05% of simulations), was increased by factors of 8.6 and 66.2 for 24 and 48 weeks, respectively.

### Cost-effectiveness analysis

Table 2 shows estimated Ebola cases and deaths, as well as costs of Ebola responses and DALYs for each of the five scenarios. Under SRPs 1–3, the average number of Ebola cases was 139.8. This number would drop to 7.8, 20.2, 33.8 and 64.7 under the scenario of SRP 4 and the three delays of the implementation of SRP 4, respectively. The number of Ebola deaths would drop accordingly. The DALYs under SRPs 1–3 were estimated to be 2311.2 versus 129.6 under SRP 4 due to the substantial reduction in the number of Ebola cases and deaths.

**Table 2:** Cost and effectiveness of Ebola response for the five scenarios

Mean of parameters	SRP 1–3	SRP 4	SRP 4 delayed 12 weeks	SRP 4 delayed 24 weeks	SRP 4 delayed 48 weeks
	(1)	(2)	(3)	(4)	(5)
Ebola cases	139.8	7.8	20.2	33.8	64.7
Ebola deaths	93.3	5.2	13.5	22.6	43.1
Active patches	22.4	4.0	6.7	9.2	14.5
Watching patches	3.9	1.3	1.8	2.4	3.4
Duration of epidemic (months)	8.2	5.3	6.4	6.9	7.7
Cost of rapid response team (million)	$39.22	$2.35	$5.18	$9.00	$19.77
Cost of contact tracing and surveillance (million)	$0.62	$0.03	$0.09	$0.15	$0.29
Lab costs (million)	$0.59	$0.03	$0.08	$0.14	$0.27
Treatment costs (million)	$1.47	$0.08	$0.21	$0.36	$0.68
Vaccine costs (million)	$1.46	$0.08	$0.21	$0.36	$0.68
Costs of psychosocial support (million)	$1.20	$0.07	$0.17	$0.29	$0.56
Cost of various preventive measures (million)	$17.52	$0.32	$1.07	$2.29	$6.06
Costs of security and preparedness (million)	$0.00	$0.52	$3.06	$4.84	$7.72
Full costs and 95% credibility intervals (million)	$62.08($0.80–$715.48)	$2.49($0.83–$18.62)	$10.07($0.95–$61.79)	$17.42($0.94–$137.42)	$36.03($0.92–$328.33)
YLD	8.0	0.4	1.1	1.9	3.7
YLL	2303.3	129.1	332.5	557.4	1064.4
DALYs and 95% credibility intervals	2311.2(0.1–25 246.7)	129.6(0.1–742.5)	333.6(0.1–2080.8)	559.3(0.1–4353.3)	1068.1(0.1–9342.0)

DALYs, disability-adjusted life years.

Since the costs for most interventions were directly associated with the number of Ebola cases, rapid control of outbreaks would significantly reduce the overall costs of Ebola response. The total cost under SRPs 1–3 was estimated to be $62.08 million ($0.80–715.48 millions) while the cost was $3.49 million ($0.83–$18.62 million) under SRP 4. The cost for security and preparedness was estimated to be $0.52 million under SRP 4. Similarly, the total cost for other SRP 4 related scenarios was somewhat lower compared with the base scenario of SRPs 1–3.

Online supplemental table S3 shows the incremental cost and effectiveness of the different response strategies. Although there was a wide variation of the incremental costs and DALYs averted, the point estimate showed a dominant effect of SRP 4 and the potential delays in implementing SRP 4.

## Discussion

### Findings

We have evaluated the epidemiological and economic impact of different strategies to respond to the 10th EVD outbreak in DRC. Despite moderate transmissibility of EVD in this outbreak and slow but steady spatial spread, it is doubtful the initial strategy would have controlled the epidemic within reasonable time. Our results suggest that when the multisectoral SRP 4 response was implemented, the reduction in EVD transmission increased threefold, accompanied by a 30% faster control of new waves, with an average time of 17.5 days compared with 24. The SRP4 strategy was able to take into account important lessons learnt on community engagement and the need for increased preparedness. It took advantage of the systems put in place during SRPs 1–3, providing more favourable conditions (*eg*, more local experts), as well as logistics support already deployed and increased community trust.

Our simulations suggest that an SRP4-like model, involving standby capacity with prioritisation of early detection, early response and community engagement, would result in smaller outbreaks than under previous strategies, in terms of caseload and spatial spread. We also show that the SRP 4-like model is most beneficial when deployed early. However, the simulated outbreak size distribution was highly skewed, with many small outbreaks (less than 10 cases), and rarer, much larger outbreaks (100s to 1000s of cases), in line with historical data.^1 8 16^

Consistent with previous analyses of EVD response in Sierra Leone,^17^ our cost-effectiveness analysis suggests that early intervention and investing in preparedness would not only have great potential to save lives, but also limit resources invested in a response.

### Limitations

A number of methodological limitations apply to this study. First, it is based on an outbreak which happened in a very specific context where insecurity played a key role driving transmission.^3^,^5^ While the transmission parameters estimated here are broadly in line with the natural history of EVD,^16^ future epidemics may differ, so caution should be taken in extrapolating our findings.

We made simplifying assumptions regarding EVD transmission and costing. We assumed a constant reporting rate throughout the outbreak, while disruptions of surveillance occurred during insecurity events. Unfortunately, estimating case reporting probabilities and their spatio-temporal fluctuations was not feasible in this outbreak for lack of sufficient data.^12^ We expect unaccounted-for changes in reporting would lead to additional variation in case incidence and result in increased uncertainty (and wider posterior distributions) in estimates of *R*. We also modelled the spatial spread of EVD through *ρ*, which measured the diffusion of the force of infection across all health zones, not accounting for potential heterogeneities in spatial connectivity. The finding that about 4.4% transmissions happened across different health zones likely reflects a mixture of higher rates of transmission across closely related health zones, and very low transmission rates between distant locations.

When comparing cost-effectiveness of different strategies, we should highlight that costs of the initial capacity to respond (*eg*, training, field laboratories, treatment centres, ultra-cold chain) were not included in SRP 4, except for maintenance and setting up of new bases. The relative cost-effectiveness of SRP 4 compared with SRPs 1–3 can be ascribed, in part, to the fact that it benefited from previous efforts. We also modelled the switch from SPRs 1–3 to SRP 4 as an instantaneous shift in efficacy for practical reasons, when in reality changes in interventions (scaling up logistics, gaining the trust of communities) were much more gradual.

Our prospective simulation study did not explicitly account for insecurity events, which could result in temporary flare-ups of transmissibility and cause multiple successive waves of infection actually observed in the data (figure 1). As a result, simulated outbreaks (figure 3A,B) were substantially smaller than the actual outbreak, as affected patches would most likely experience a single wave of cases. We expect this general scaling down of outbreak sizes should not alter the relative differences observed in different response strategies.

Another limitation of this study is the lack of resolution into different aspects of interventions. Indeed, we contrasted strategies, without detailing activities such as contact tracing, vaccination or community engagement, and their intensities over time. While such insights would undoubtedly be invaluable, modelling the relative impact of different activities on transmission, and their interplay, would lead to a much more complex, over-parameterised model, which could not be fitted with available data. The cost-effectiveness analysis suffers from similar limitations, as we were unable to distinguish capital costs from recurrent costs for Ebola response, so that the early investment in pandemic preparedness was likely underestimated. The joint cost (including indirect costs) for each strategy was estimated with the assumption that it was proportional to the scale of the outbreak. As a consequence, cost savings were a mere consequence of the reduction of transmission, the largest ones corresponding to the most expensive interventions (rapid response teams and prevention, table 2). In addition, as the outcome of cost-effectiveness was DALYs, we were not able to look at the longer term impact of this outbreak on building health systems or health infrastructure for future outbreaks. The focus of the cost-effectiveness analysis on direct health system costs excluded household costs, and therefore is expected to substantially underestimate the cost-effectiveness from a societal perspective.

### Policy recommendations

These findings can be used to inform future EVD interventions. Prioritising preparedness, with a focus on standby capacity allowing for a rapid response, appears to be an effective strategy to control EVD outbreaks. As such, SRP 1–3 interventions could be considered as preparedness activity for a more multisectoral intervention. The benefits of such a large-scale intervention are highly dependent on the scale of the epidemic at hand. With Ebola expected to cause mostly small outbreaks, and larger ones only infrequently, it is likely that strategies akin to SRPs 1–3 would suffice to contain most EVD outbreaks. But in situations of higher transmissibility, for example, due to a more virulent viral strain, high population density, or in contexts of sustained insecurity, our results suggest a rapid scale-up to SRP 4 would not only save lives but also be cost-effective.

Bolstering support for multisectoral interventions was likely critical for controlling the epidemic, in a unique context marked by insecurity and humanitarian concerns, and immense community needs. While we lack data on the relative impact of specific interventions, it seems likely that increased community engagement played a central role in controlling this outbreak, as it directly impacted several key aspects of the response, enabling faster and more comprehensive case detection, more timely isolation and treatment of patients, more complete contact tracing, and therefore more effective vaccination.

The provision of resources to reinforce the supporting environment seems particularly vital in large-scale epidemics responses, where it will strengthen key activities such as vaccination, treatment and contact tracing. Although deployment of multisectoral interventions would ideally occur early, minimising both the health and economic impact of EVD, mobilising resources and formulating comprehensive intervention plans take time, and our findings highlight that even in the presence of delays, implementing comprehensive and effective interventions continues to be cost-effective.

## Supplementary material

10.1136/bmjgh-2024-015822 online supplemental file 1

10.1136/bmjgh-2024-015822 online supplemental file 2

## Data Availability

Data may be obtained from a third party and are not publicly available.
