# Supplementary material for: Contrasting the impact and cost-effectiveness of successive intervention strategies in response to Ebola in the Democratic Republic of the Congo, 2018–2020
Source: BMJ Glob Health. 2025 Apr 15;10(4):e015822. doi: 10.1136/bmjgh-2024-015822 (PMC12004461; doi:10.1136/bmjgh-2024-015822)
Supplement: online supplemental file 1 [file bmjgh-10-4-s001.pdf]

Estimating the impact and cost-effectiveness of the response to Ebola in the Democratic Republic of the Congo, 2018-2020

Thibaut Jombart, Wu Zeng, Michel Yao, Anne Cori, Hadia Samaha , Thomas Wilkinson, Mathias Mossoko, Jean-Pierre Lokonga, Dominique Baabo, Fatima El Kadiri El Yamini, Patrick, Hoang-Vu Eozenou, Sylvain Yuma, Linda Mobula

Table of contents

Text S1: Identification of epidemic waves 2
Text S2: Epidemiological model 3
Text S3: Estimation of epidemiological parameters 4
Text S4: Cost-effectiveness analyses 5
Figure S1: estimated distributions of delays to control of the waves 6
Figure S2: MCMC traces of log-likelihoods values 7
Table S1: outline of the strategic response plans (SRPs). 8
Table S2: parameters of the distributions of delays to controlling waves. 10
Table S3. Incremental cost and DALYs averted (95% credibility intervals), for SRP 4 based strategies, compared to SRP 1-3 10

Text S1: Identification of epidemic waves

As our model aimed to estimate separate transmission parameters for phases of epidemic growth and decline, these phases were characterized for each health zone separately. To identify the different waves of infection in a given health zone, we first detected temporal clusters of cases using the *vimes* approach [11], which can be used to define clusters of related cases based on the expected distribution of distances (in time or space for example) between any two epidemiologically linked cases. Here, *vimes* was used with temporal distances only, defined as the numbers of days separating cases' symptom onsets, with a threshold of 32 days corresponding to the 95th percentile of the serial interval of Ebola [5,12]. Simply put, clusters of cases more than 32 days apart would be classified as separate waves. Results were manually refined to further distinguish successive waves which were not separated by periods of zero incidence, using local dips in incidence as an indicator of separate waves. Peaks were defined as the day of highest incidence for each wave, so that each day in each health zone could be classified into 3 categories: growth phase (before the peak of the wave), 'control' phase (i.e. after the peak) during either SRPs 1-3, or SRP 4.

Text S2: Epidemiological model

We used a discrete-time (daily) meta-population, branching process model to describe the transmission of EVD in different health zones (referred to as 'patches' in the model). In its simplest form, the model states that the force of infection (FOI) generated by infected cases in patch *k* and time *t*, denoted $\lambda_{k,t}$, is defined as [5,14]:

$$\lambda_{k,t} = \sum_{s=1}^{t-1} R_{k,s} y_{k,s} w(t-s)$$

where $R_{k,s}$ is the effective reproduction number in patch *k* at time *s*, $y_{k,s}$ is the number of new symptomatic cases in patch *k* at time *s*, and $w(.)$ is the probability mass function of the serial interval, *i.e.* the time interval between the dates of symptom onset of infectors and the secondary cases they infected.

We assume that a proportion ρ of the FOI is distributed to other patches, assuming uniform dispersal across all other patches, so that the FOI of patch *k* onto itself at time *t* is calculated as:

$$\lambda_{k \to k,t} = (1-\rho)\lambda_{k,t}$$

and the FOI to other patches at time *t* is defined as:

$$\lambda_{k \to l, l \neq k, t} = \frac{\rho}{K-1}\lambda_{k,t}$$

where *K* is the number of patches.

The FOI experienced by patch *k* at time *t* is the sum of all FOIs directed towards this patch:

$$\gamma_{k,t} = \sum_{m=1}^{K} \lambda_{m \to k,t}$$

And we assume that the number of new symptomatic cases generated in patch *k* at time *t*, conditional on previous incidence, transmission and dispersion, is governed by a Poisson process:

$$y_{k,t} \sim Poisson(\gamma_{k,t})$$

Because it is not feasible to estimate different reproduction numbers every day in every patch, we simplified the model so that $R_{k,s}$ could take 3 possible values, depending on the phase in which a patch *k* is on day *s*. During a growth phase we set $R_{k,s} = R$, where *R* is the effective reproduction number before control of a wave. The impact of intervention is modeled through the intervention efficacy ε, defined as the relative reduction of transmission during control phases. We estimate separate efficacies for the SRP1-3 and SRP4 strategies, so that we have $R_{k,s} = (1 - \varepsilon_{SRP1\text{-}3})R$ if patch *k* is in a control phase during the SRP1-3 strategy at time s, and $R_{k,s} = (1 - \varepsilon_{SRP4})R$ for control during the SRP4. Since the overall number of cases in the outbreak remained negligible compared to the population size of the affected area, the impact of the depletion of susceptibles was not included in the model.

Text S3: Estimation of epidemiological parameters

Our model uses data on daily new symptomatic cases stratified by health zones ('patches'), denoted *y*. We used published estimates for the serial interval distribution, parametrized as a discretized Gamma distribution with mean 15.3 days and standard deviation 9.3 days [12]. We used a Bayesian Markov Chain Monte Carlo (MCMC) framework to explore the distributions of the 4 parameters of the model: R, ρ, $\varepsilon_{SRP1\text{-}3}$ and ε_{SRP4}. The likelihood of the model is defined as:

$$p(y|R,\rho,\varepsilon_{SRP1-3},\varepsilon_{SRP4}) = \prod_{t=1}^{T} p(y_t|y_{s<t},R,\rho,\varepsilon_{SRP1-3},\varepsilon_{SRP4}) = \prod_{t=1}^{T}\prod_{k=1}^{K} f(y_{k,t},\gamma_{k,t})$$

where T is the total number of days in the outbreak and $f(a,b)$ denotes the probability mass function of the Poisson distribution for '*a*' events and a rate '*b*'.

Because of the rather exceptional setting of the outbreak in North Kivu / Ituri, where insecurity had a key impact on EVD transmission [1,3–5], we avoided using informative priors for transmission parameters, and instead assumed flat priors with uniform distributions covering all plausible values for R ($U(0,10)$, and all possible values for ρ, $\varepsilon_{SRP1\text{-}3}$ and ε_{SRP4} ($U(0,1)$)). As a consequence, the posterior distribution is directly proportional to the likelihood function described above. The Metropolis algorithm was used to explore the parameter space using Normal proposal distributions for all parameters, with a standard deviation of 0.02 for R and of 0.01 for all other parameters. 12 independent chains were run in parallel to assess mixing and increase posterior sample size. Each chain was run for 5,000 iterations, burn-in was assessed visually and the corresponding first 100 iterations discarded, and samples were selected every 50 steps of the remaining iterations. In the end the posterior sample size was 1,176.

Text S4: Cost-effectiveness analyses

The numbers of cases, deaths, and active patches of our simulations were used to estimate the corresponding costs for each simulation. Table 1 shows the costs associated with each of the key health interventions in managing the Ebola response during 2018-2020. The cost of the intervention was estimated using the unit costs in Table 1 multiplied by the corresponding quantity. For example, the laboratory test cost was assumed to be US$1,464 per identified EVD case. Then the total laboratory cost was estimated to be the product of the number of EVD cases and US$1,464.

Some services, such as psychosocial support and vaccination, were not directly expressed as costs per EVD case. For these, we used the ratios of number of people who received these services to the corresponding EVD cases to convert costs into per EVD case units. For example, we calculated that 49.24 individuals on average received psychosocial support per EVD case. We would thus estimate the size of the population who would need psychosocial support to be 49.24 times the number of EVD cases in a given simulation, and derive costs accordingly. For interventions whose costs were a function of the duration of Ebola outbreaks, such as rapid response team deployment, prevention, coordination, community engagement, operation monitoring and evaluation, we used the duration of each simulated outbreak (assuming outbreaks ending after three consecutive months with no EVD case) to calculate the associated costs. The total cost for the5 scenarios described above was generated by summing up the costs across the interventions.

The number of EVD cases and the number of deaths from EVD generated from each simulation were used to estimate disability adjusted life years (DALYs) for each scenario. DALY is the sum of the life years lost due to disability (YLD) for non-fatal cases and the life years lost due to premature deaths (YLL) for fatal cases. To calculate YLD for the non-fatal EVD cases, a disability weight of 0.133 (0.088-0.190) and the duration of an EVD episode of 15.1 days (14.6-15.6) were used [15]. Additionally, we consider the post-Ebola chronic fatigue for non-fatal EVD cases, with a disability weight of 0.219 (0.198-0.414) and a duration of 0.75 years (0.417-1.135) [16]. For each fatal case, we use the following formula to calculate YLL [17].

$$YLL = (1 - e^{-r*L})/r$$

where r is the discount rate, for which we used 3% in the study, and L is the life expectancy at the age of death from EVD. Based on the data from Médecins Sans Frontières (MSF) derived from 724 confirmed EVD cases, we estimated that the average age of death of EVD fatal cases is 19.2 years old (calculated from a database from DRC), with 45 years of life expectancy based on the life table for DRC [18]. The total DALYs were calculated for the 5 scenarios.

Using the scenario of SRP 1-3 as status quo, we compared the incremental costs and the incremental effectiveness (DALYs averted). Given that the results showed a dominant effect of SRP 4 and the various delays in implementation of SRP 4, we did not generate incremental cost-effectiveness ratios (ICERs) for the SRP 4 related scenarios.

Figure S1: estimated distributions of delays to control of the waves

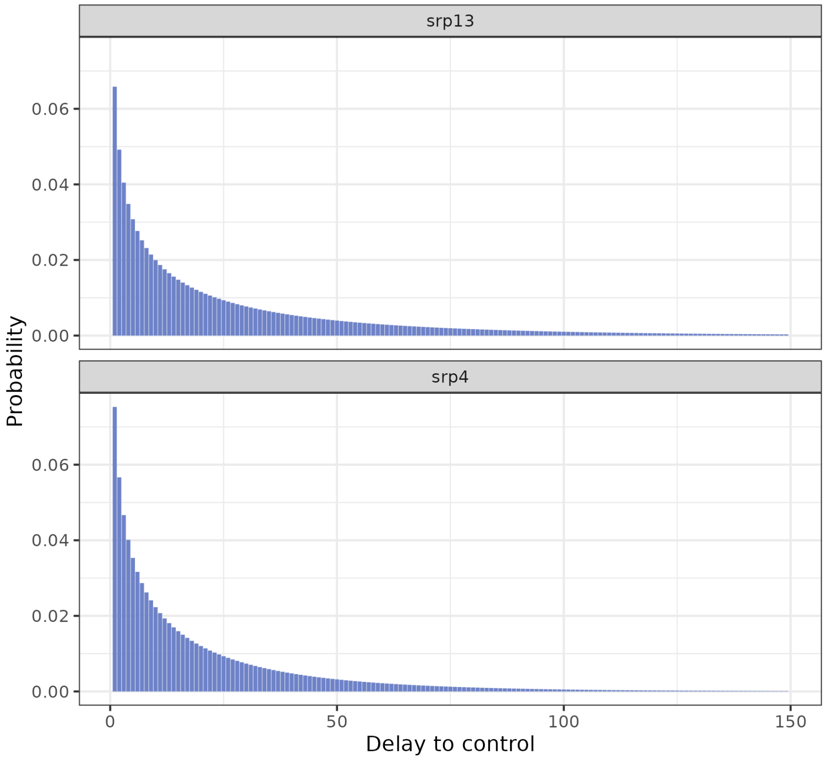

This figure shows the probability mass function of two discretized Gamma distributions fitted to empirical delays to control the different waves of EVD in the outbreak, during the SRP 1-3 (top panel) and the SRP 4 (bottom panel). Fitting was achieved using the Maximum-Likelihood procedure implemented by the function fit_disc_gamma from the R package *epitrix*[*(Small Helpers and Tricks for Epidemic...)*]. See Table S1 for parameter values.

Figure S2: MCMC traces of log-likelihoods values

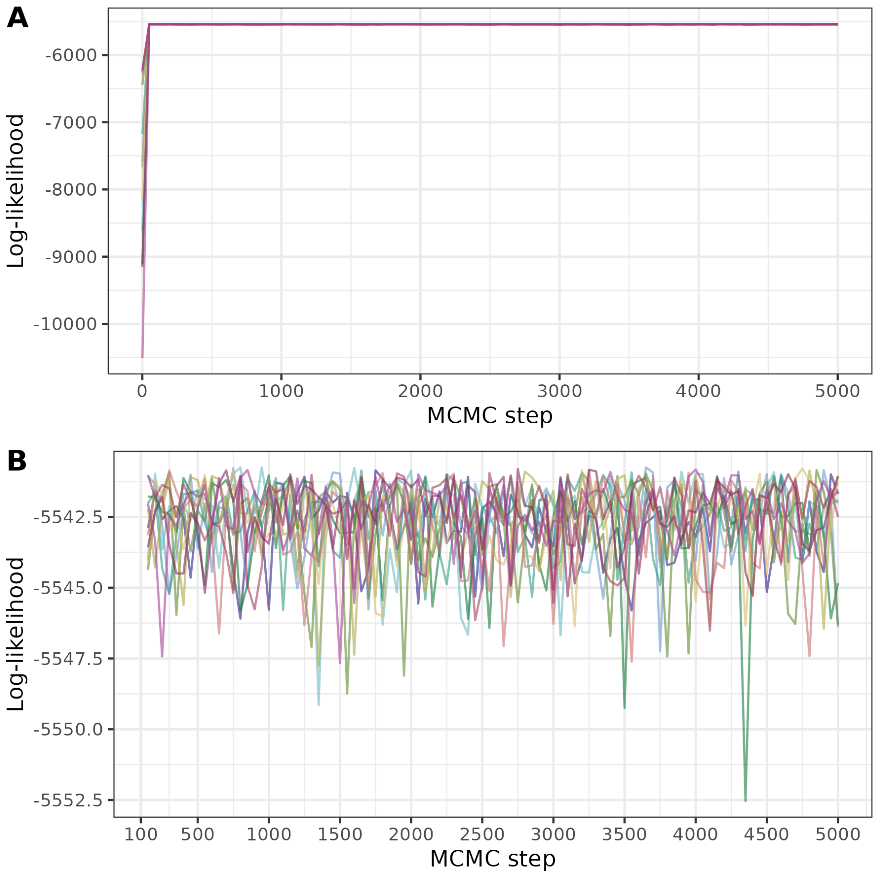

These plots show the values of log-likelihood corresponding to the sampled parameters across the different MCMCs. Colors identify 12 independent chains run in parallel, each for 5,000 iterations, sampled every 50 iterations. A) Full traces. B) After discarding the first 100 steps as burn-in. Estimation of the model parameters was achieved by MCMCs using the Metropolis algorithm to explore the parameter space.

Table S1: outline of the strategic response plans (SRPs).

SRP	Context	Time frame	Overall response strategy	Response areas
**SRP 1**	Cluster of cases reported late in six health zones (districts) with multiple contacts of cases not tracked, in a conflict and humanitarian context without any local Ebola response experience.	August 2018 to October 2018	Immediate surge of available solutions (treatment and vaccination innovations) and experienced experts to catch up delayed response with weak local resources and health system.	● Coordination<br>● Surveillance<br>● Vaccination<br>● Clinical care<br>● Laboratories and research including field laboratories<br>● Infection prevention and control; safe and dignified burial<br>● Risk communication and community engagement<br>● Psychosocial care<br>● Logistic and administrative support<br>● Security<br>● Training of rapid response teams
**SRP 2**	Further geographical spread; change in the operating environment with limited access to communities for critical response interventions due to insecurity and resistance to those interventions.	November 2018 to January 2019	Scale up response capacity to cover a wider area of response operations including greater Congolese government and partners contributions (cover ten additional health zones); still a surge in areas with no response capacity; greater involvement of humanitarian actors for humanitarian needs to improve acceptance of Ebola response	Scaling up different response areas started with SRP-1, based on lessons learnt with a greater contribution of social scientists and further capacity building (local entities and partners)
**SRP 3**	Significant increase in new cases; political challenges (with increased demonstrations as Eastern regions excluded from elections in December 2018) including increased violence against civilians and Ebola responders; non-compliance to control measures hampering some interventions like contact tracing (reached 30% of new cases known as contact around April 2023).	February 2019 to June 2019	Increase field presence (response decentralization) to adapt to local challenges and speed up response by rapid response teams (aiming at deployment of response teams to newly affected areas within 48h) with further spread of the disease in health zones and population movements due to insecurity avoiding control measures, a major regional threat. Further Integration of social scientists into the epidemiological analytics cell for better analysis and interventions in hot spots with rapid response teams. Strengthening community engagement (interventions by trained community members). Set up an accountability and responsibility framework with further involvement of the existing health system. Tackled lost contacts and strengthened other health services (child and maternal health) while ensuring readiness in areas not yet affected, putting all health zones in response mode.	Building on SRP-1 and SRP-2 response areas based on lessons learnt with additional preparedness activities in neighboring countries and provinces.

**SRP 4**	SRP-3 by June impacted on the two major hot spots (Katwa and Butembo) but with new areas affected, increased insecurity and community resistance hampering the response. High regional risk with cases in Uganda. Communities calling for more interventions to address their comprehensive basic needs in a context of extremely low funding levels for humanitarian interventions.	July 2019 to December 2019	Focus on rapid detection, isolation with a rapid multisectoral interventions around confirmed cases. Responsibility of each response area assigned to national entities and organizations with related comparative advantages.	Building on SRP1, SRP-2 and SRP-3 response areas based on lessons learnt with more dimensions added to the public health response: political and community engagement; security and operation support; support to communities; an integrated financial planning; monitoring and reporting; preparedness in surrounding countries.
**SRP 4.1**	Reduction of new cases<br>During the last 4 weeks of December 2019 but still volatile and need to further build local capacity and start the transition phase.	January 2020 to June 2020	Last step toward controlling the outbreak with targeted strategy based on the epidemiological situation around scenarios. Transition towards local capacity building and strengthening of local health systems, scaling up of other basic health services.	Building on SRP1, SRP-2, SRP-3, SRP-4 response areas based on lessons learnt with transfer of competences to local health entities in response areas and ensuring a stronger community involvement.

Table S2: gaps in strategic response plans (SRPs).

SRP	Time frame	Outline of gaps and strategic decisions
**SRP 1**	August 2018 to October 2018	With late detection of the outbreak, health worker infections, and hospital contamination, an urgent scale-up was necessary. Experienced staff were rapidly deployed to ensure an effective response.
**SRP 2**	November 2018 to January 2019	As non-compliance, population displacement, and multiple hotspots emerged, the strategy focused on decentralizing teams (including training local staff) and engaging communities to reduce resistance.
**SRP 3**	February 2019 to June 2019	Faced with low contact tracing, mobile populations, and worsening community resistance and violence, the strategy emphasized scaling up efforts, rebuilding trust, and improving compliance with control measures.
**SRP 4 and 4.1**	July 2019 to June 2020	With enhanced capacity and decentralized teams in hotspots, an integrated approach emerged. In a challenging (growing insecurity), underfunded humanitarian context, SRP 4 created an enabling environment, prioritized local implementation and community involvement ("faire faire"), and introduced innovations such as cantonment for contacts in insecure areas who requested assistance, with more effective integrated interventions.

Table S2: parameters of the distributions of delays to controlling waves.

	Mean	Standard deviation	Coefficient of Variation	Shape	Scale
**SRP 1-3**	**24.0**	**34.4**	**1.43**	**0.4883**	**49.198**
**SRP 4**	**17.5**	**24.3**	**1.39**	**0.520**	**33.657**

Table S3. Incremental cost and DALYs averted (95% credibility intervals), for SRP 4 based strategies, compared to SRP 1-3

	Incremental cost (Million)	DALYs averted
SRP 4 vs SRP1-3	-$58.60 (-$708.34-$14.41)	2,181.7 (-594.7-25,162.1)
SRP 4 delay 12 weeks vs. SRP 1-3	-$52.01 (-$700.82 - $55.56)	1,977.6 (-1,882.4-25,050.6)
SRP 4 delay 24 weeks vs. SRP 1-3	-$44.66 (-$703.24-$120.89)	1,752.0 (-3,963.4-24,827.8)
SRP 4 delay 48 weeks vs. SRP 1-3	-$26.05(-$688.83-$312.746)	1,243.2(-8,828.7-24.580.5)
