# Supplementary material for: Contrasting the impact and cost-effectiveness of successive intervention strategies in response to Ebola in the Democratic Republic of the Congo, 2018–2020
Source: BMJ Glob Health. 2025 Apr 15;10(4):e015822. doi: 10.1136/bmjgh-2024-015822 (PMC12004461; doi:10.1136/bmjgh-2024-015822)
Supplement: online supplemental file 2 [file bmjgh-10-4-s002.docx]

**Reflexivity statement**

1. How does this study address local research and policy priorities?

EVD epidemics pose a constant threat to the DRC. Research on response efficacy is therefore an important Public Health contribution and will serve to provide direction on policies on Health Security, especially as DRC is updating its National Action Plan for Health Security.

2. How were local researchers involved in study design?

MM was leading all data-analytics aspects of the Ebola response for the Ministry of Health of the DRC. SAM, JPL, DB, SY assisted with study design and interpretation of results. JPL assisted with data collection.

3. How has funding been used to support the local research team?

World Bank Funding was used to support salaries for JPL and DB.

4. How are research staff who conducted data collection acknowledged?

Data was collected in the field as part of the EVD response operations. The data was collated and curated by MM.

5. Do all members of the research partnership have access to study data?

All members of the partnership have access to the data.

6. How was data used to develop analytical skills within the partnership?

During the EVD response, TJ and MM organized regular in-country training and skill-sharing workshops for all team members. Analyses were conducted jointly by local and international staff.

7. How have research partners collaborated in interpreting study data?

The interpretation of the data and results were conducted jointly by local and international staff.

8. How were research partners supported to develop writing skills?

The research team is composed of senior academics and public health agency staff, so that no particular writing support was needed.

9. How will research products be shared to address local needs?

The research described in this piece will serve as the foundation for the development of a free, open-source tool for evaluating the impact and cost-effectiveness of intervention strategies, which will be shared with the DRC Ministry of Health.

10. How is the leadership, contribution and ownership of this work by LMIC researchers recognised within the authorship?

The authorship acknowledges the key role of SAM, MM, JPL, DB, SY in designing data collection, and subsequently collating and curating the data, driving the original objectives of the research, and operationalising the results.

11. How have early career researchers across the partnership been included within the authorship team?

This research does not involve early career researchers.

12. How has gender balance been addressed within the authorship?

4 authors (AC, HS, FEKEY, LM) are female and 10 authors are male (TJ, WZ, SAM, MY, TW, MM, JPL, DB, PHVE, SY) ; the senior author, leading the project (LM), is female.

13. How has the project contributed to training of LMIC researchers?

The original modelling work underpinning this project was carried in-country by TJ during the EVD response, and was presented and discussed at multiple occasions with the response team led by MM.

14. How has the project contributed to improvements in local infrastructure?

This project has not directly contributed to improvements in local infrastructure, but will serve as a basis for a software tool which will be of direct use to public health officers in the DRC.

15. What safeguarding procedures were used to protect local study participants and researchers?

There was no primary data collection as part of this project, therefore this question is not directly applicable. The data we used was secondary data (aggregated case incidence), derived from primary data collected as part of the response to an international health emergency.
